# Insulin-like growth factor-1 (IGF-1) empowering tendon regenerative therapies

**DOI:** 10.3389/fbioe.2025.1492811

**Published:** 2025-03-27

**Authors:** Mingming Wang, Jiayuan Zhang, Hanyue Li, Yini Li, Zhigang Li

**Affiliations:** ^1^ Police Basic Skills Teaching Department, Sichuan Police College, Luzhou, Sichuan, China; ^2^ School of Physical Education, Southwest Medical University, Luzhou, Sichuan, China; ^3^ The Affiliated Hospital of Southwest Medical University, Luzhou, Sichuan, China

**Keywords:** tendon injury, IGF-1, tendon regenerative healing, tendon repair, tenocyte, mechanisms

## Abstract

Tendon injury is one of the most common musculoskeletal disorders that severely affect patients’ daily lives. Unfortunately, naturally healed tendons exhibit poor quality, as they have very limited regenerative ability. Recently, therapeutic strategies involving the administration of growth factors have been advocated to enhance tendon regenerative healing. Growth factors are peptide-signaling molecules that elicit biological functions such as cell proliferation, migration, and differentiation by acting through a complex organization of cell surface receptors and activating intracellular signaling pathways. Insulin-like growth factor-1(IGF-1) represents one such factor that has shown promising effects for enhancing tendon regenerative healing *in vitro* and animal models. However, it is disappointing that IGF-1 has not been shown to play a very significant role in promoting tendon healing in clinical trials, which could reflect our poor understanding of the molecular mechanisms by which IGF-1 is involved in promoting tendon regenerative healing. Therefore, in this review, we summarized the roles and mechanisms of IGF-1 for enhancing tendon regenerative healing. Nevertheless, much work is still needed to optimize its effectiveness.

## 1 Introduction

Tendon injury is a common and disabling musculoskeletal condition, accounting for over 30% of all musculoskeletal consultations (Wang et al., 2019a). Unfortunately, the innate limited spontaneous regenerative capability of tendons poses a substantial treating challenge for clinicians as the healed tendons often heal through the formation of scar tissue with the inferior biomechanical and biological properties compared to the intact tendon tissue (Li S. et al., 2023). Current therapeutic strategies for tendon injury can be broadly divided into operative or conservative treatments (Adjei-Sowah et al., 2023). When conservative treatments fail, treatments frequently resort to surgical treatment (Liang et al., 2024). Unfortunately, both conservative and surgical approaches for managing tendon injuries present significant limitations (Silbernagel et al., 2020). Conservative treatment typically requires prolonged healing periods, ranging from 4 to 6 weeks for mild injuries, 6–12 weeks for moderate cases, and up to 3–6 months or longer for severe injuries (Kosiol et al., 2023). Similarly, surgical intervention entails an extended recovery timeline, with an average of 6–8 weeks for initial wound healing and several months for full functional restoration (Reda et al., 2020). In terms of re-rupture rates, conservative treatment is associated with a risk of 10%–30%, influenced by factors such as injury type, severity, and patient adherence to rehabilitation protocols (Seow et al., 2023). Although surgical treatment demonstrates a comparatively lower re-rupture rate (5%–15%), it still poses a substantial clinical challenge. Given these limitations, there is an urgent need to develop novel therapeutic strategies to improve outcomes in tendon injury management.

Recent studies have highlighted the potential of various growth factors in promoting tendon regenerative healing. Preclinical research has demonstrated that factors such as vascular endothelial growth factor (VEGF), fibroblast growth factor-2 (FGF-2), transforming growth factor-β1 (TGF-β1), and insulin-like growth factor-1(IGF-1) may contribute to tendon repair (Lai et al., 2022; Guo et al., 2020; Deng et al., 2024). However, their therapeutic applications are limited by significant drawbacks. For instance, VEGF has been shown to disrupt the extracellular matrix (ECM) of tendon tissues (Liu et al., 2021), while TGF-β1 is associated with the formation of adhesions and scar tissue, leading to fibrosis and restricted tendon mobility (Li H. et al., 2023). Furthermore, exogenous administration of FGF-2 in a canine model failed to improve the biomechanical properties of injured tendons (Thomopoulos et al., 2010).

Compared to other growth factors, compelling evidence suggests that IGF-1 plays a pivotal role in enhancing the regenerative potential of injured tendons. For instance, Kurtz et al. demonstrated that exogenous IGF-1 administration reduces functional deficits and accelerates recovery in a rat Achilles tendon injury model (Kurtz et al., 1999). *In vitro* studies further support its therapeutic potential, showing that IGF-1 modulates inflammatory responses, stimulates tenocyte proliferation and migration, and enhances collagen synthesis (Gulotta and Rodeo, 2009; El Essawy and Baar, 2023). However, despite its success in preclinical models, clinical trials have shown limited efficacy of IGF-1 in promoting sustained recovery in patients with tendinopathy (Olesen et al., 2021). These discrepancies may stem from an incomplete understanding of the mechanisms underlying IGF-1’s actions during tendon healing. In this review, we aim to summarize the roles and mechanisms of IGF-1 in tendon regenerative healing, providing insights into its potential clinical applications for patients with tendon injuries. Nevertheless, further research is essential to optimize its therapeutic effectiveness and translate preclinical findings into clinical success.

## 2 Materials and methods

**FIGURE 1:**
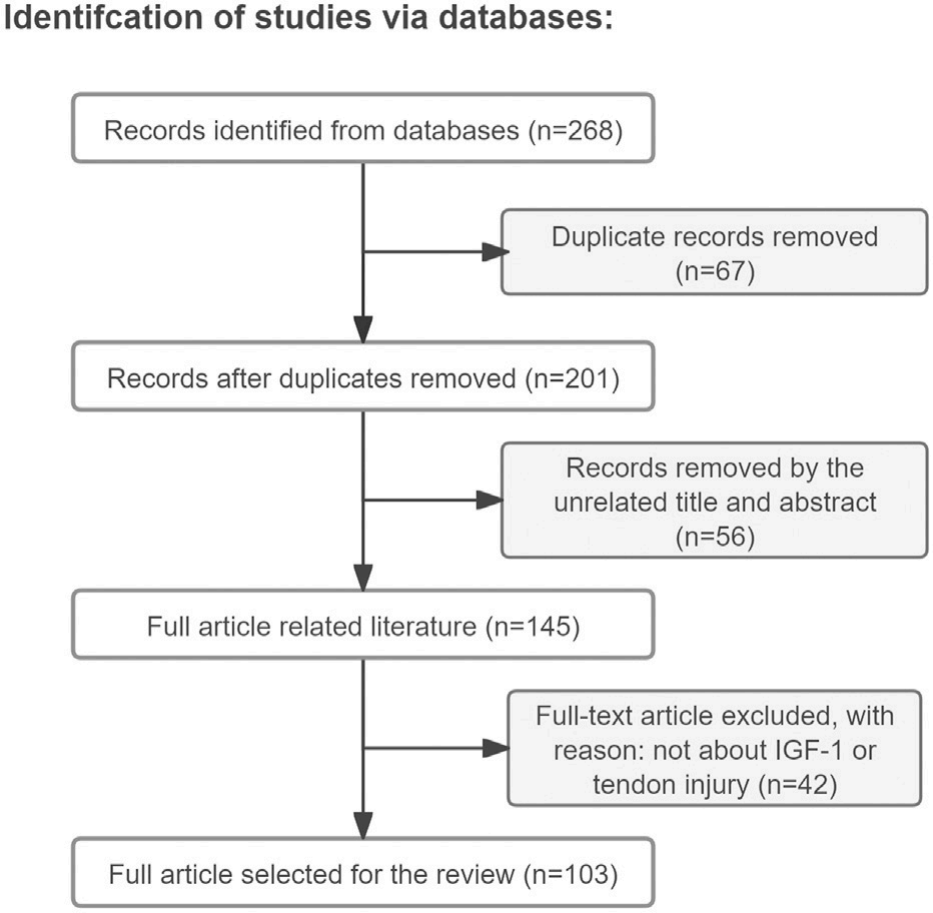
Article retrieval flow chart with inclusion and exclusion process.

Approximately 90% of the papers mentioned in this review (total number of papers included in this review: 103) were found by a search in the Web of Science/Google Scholar databases using the keywords “tendon injury, IGF-1, tendon regenerative healing, tendon repair, tenocyte, mechanisms” (the search was performed during the period of May to September 2024 and only literature written in English was reviewed) (Figure 1). The remaining 10% were cited in the references found within this search, with the exception of some very recent papers. The reviewed literature was published from 1988–2024. Original research papers and review papers were included. We focused on IGF-1 administration to tendon injury, either by single injection or released in a sustained manner from a delivery device. Additionally, full transections of tendons, partial lacerations, and tendinopathy animal models, such as collagenase-induced tendinopathy, were included and the effects and mechanisms of IGF-1 on the healing process were discussed.

## 3 IGF-1 and its signal transduction pathway

IGF-1, organically named somatomedin C, is a multifunctional peptide mitogen possessing a molecular weight of 7,649 daltons (Geffken et al., 2022). It consists of 70 highly conserved amino acids in a single chain with three intramolecular disulfide bridges, with high structural homology with insulin (Al-Samerria and Radovick, 2021). IGF-1 is synthesized in various tissues like the liver, skeletal muscle, bone, and tendon (Lygren et al., 2013). Significantly, the liver is the main source, accounting for 80% of IGF-1 synthesis and secretion. The remaining 20% is synthesized locally, often by connective tissue cells (Roberts et al., 2023).

**FIGURE 2:**
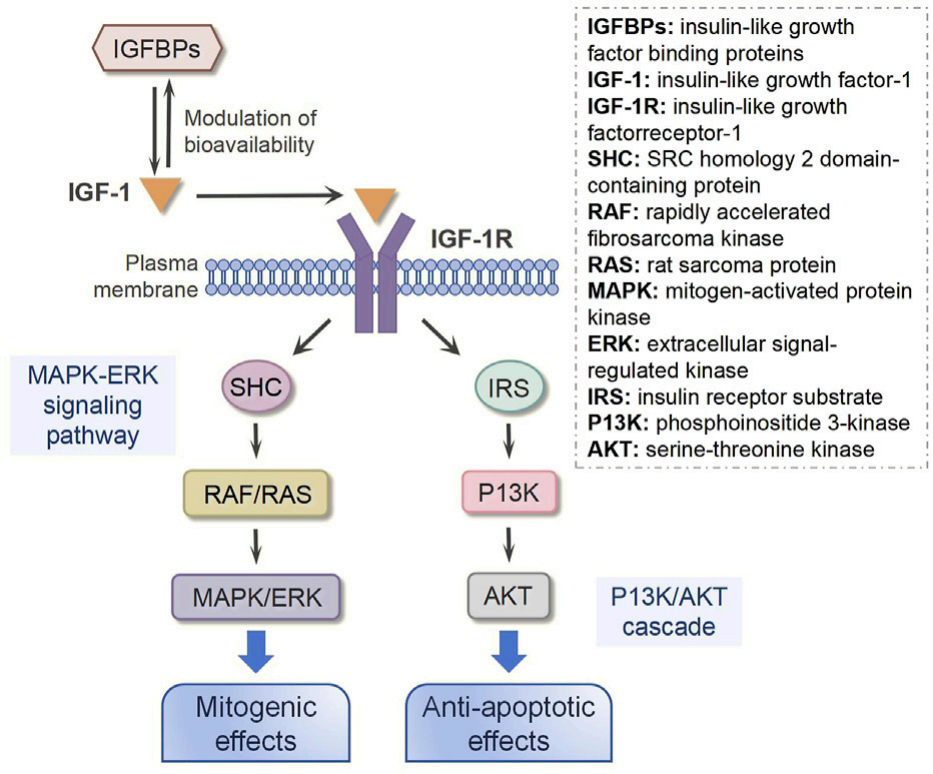
IGF-1 signaling transduction.

Following either local secretion or systemic circulation to target tissues, IGF-1 primarily exerts its biological effects through binding to the IGF-1 receptor (IGF-1R), with minimal interaction occurring through the insulin receptor (IR) (Figure 2) (Werner, 2023). The IGF-1R, which is ubiquitously expressed across nearly all cell types in the organism, consists of two α and two β subunits linked by disulfide bonds (Bailes and Soloviev, 2021). The β subunits contain intracellular tyrosine kinase domains that are activated upon IGF-1 binding to the extracellular α subunits, initiating downstream signaling cascades (Muraguchi et al., 2019). Ligand binding to IGF-1R triggers tyrosine kinase domain activation, resulting in autophosphorylation of tyrosine residues (Rieger and O'Connor, 2020). This phosphorylation event subsequently activates various substrates, including insulin receptor substrate (IRS) and SRC homology 2 domain-containing protein (SHC), which in turn recruit additional signaling molecules to activate two major intracellular pathways: the phosphoinositide 3-kinase (PI3K)/serine-threonine kinase (AKT) pathway and the mitogen-activated protein kinase (MAPK)/extracellular signal-regulated kinase (ERK) pathway (Wang J. et al., 2023; Scott et al., 2005). The MAPK/ERK signaling cascade primarily mediates cellular proliferation, while the PI3K/AKT pathway predominantly regulates cell survival and apoptosis (Macvanin et al., 2023; Matsushita et al., 2022). Notably, research has demonstrated that ginsenoside Rg1-mediated activation of IGF-1R and MAPK signaling pathways significantly enhances tendon healing in a rat Achilles tendon injury model, underscoring the crucial role of these signaling pathways in tendon repair processes (Wu et al., 2022).

In addition to IGF-1R, insulin-like growth factor binding proteins (IGFBPs) are also required for IGF-1 bioavailability because they interact with IGF-1 with the same affinity as IGF-1R (Perks, 2023). IGFBPs are synthesized by various cell types, including hepatocytes, fibroblasts, and osteoblasts (Zerti et al., 2021). As primarily secreted proteins, IGFBPs are released into the extracellular space, where they interact with IGF-1 (Oxvig and Conover, 2023). These proteins can circulate in the bloodstream or reside in tissue interstitial fluid, enabling them to bind freely circulating IGF-1 or IGF-1 associated with cell-surface receptors (Wang et al., 2021). Interestingly, emerging evidence indicates that IGFBPs can also localize to the cell membrane, where they may interact with membrane receptors or other surface-associated proteins (Tian et al., 2024). This membrane-bound form of IGFBPs plays a regulatory role in IGF-1 signaling, either by modulating IGF-1 binding to its receptors or by initiating distinct signaling pathways, thereby fine-tuning cellular responses (Zhang et al., 2023). It has been reported that IGFBPs can bind approximately 98% of all circulating IGF-1, and their binding affinity for IGF-1 is nearly 10 times higher than that for IGF-1R (Zhong et al., 2023). Thus, the function and concentration of IGFBPs are critical to regulating the biological action of IGF-1. In a rat Achilles tendon injury model, Western blot and immunobiological staining results showed more IGF-1 in the injured tendon following local administration of IGFBP-4. Downstream of IGF-1, AKT was activated and phosphorylated, subsequently promoting tendon healing (Wang H. et al., 2023).

## 4 Tendon scar healing

Following a tendon injury, the healing process unfolds through three overlapping phases: (1) the inflammatory phase, (2) the proliferative phase, and (3) the remodeling phase (Leong et al., 2020). The inflammatory phase initiates within minutes post-injury and persists for several days, marked by the dynamic recruitment of immune cells. These cells secrete a cascade of proinflammatory cytokines and growth factors, including VEGF, TGF-β, and IGF-1, which amplify the inflammatory response (Alim et al., 2020). Subsequently, the proliferative phase commences, lasting several weeks. During this stage, there is a significant increase in ECM production—primarily type III collagen—and cellularity, predominantly tenocytes (Leahy et al., 2023). Macrophages transition from a proinflammatory to a reparative phenotype, releasing growth factors and chemoattractants that promote tenocyte proliferation and recruitment (Li H. et al., 2023). The remodeling phase begins 1–2 months after injury and can extend beyond a year. This phase is characterized by a shift toward collagen type I synthesis and improved ECM alignment (Li et al., 2024). Concurrently, tenocyte density and synthetic activity gradually decline. Despite these repair mechanisms, the healing process often results in fibrotic tissue formation around the injury site, characterized by disorganized and excessive ECM deposition. This fibrotic tissue is mechanically inferior to native tendon and contributes to functional impairment (Korcari et al., 2022).

## 5 The expression profile of IGF-1 in tendon healing

A controlled delivery strategy for IGF-1, which accounts for the spatiotemporal complexity of the injury microenvironment and synergizes with endogenous IGF-1 production through feedback mechanisms, holds significant promise for enhancing IGF-1 therapy (Prabhath et al., 2018). Therefore, understanding the endogenous expression profile of IGF-1 during tendon healing is crucial for determining the optimal timing of exogenous IGF-1 administration. In the following sections, we review studies investigating IGF-1 expression during tendon healing in animal models, categorized based on the timing of IGF-1 expression peaks.

In the early-peaked studies, Hansson et al. observed a transient increase in IGF-1 expression at day 3 following vibration-induced Achilles tendon injury in rats (Hansson et al., 1988). Similarly, in a chicken flexor tendon repair model, IGF-1 expression was significantly upregulated at day 3 but returned to baseline levels by day 9 and remained stable through day 21 (Chen et al., 2008).

In late-peak studies, In a rat supraspinatus tendon injury model induced by overloading, IGF-1 immunostaining was minimal at 4 and 8 weeks but markedly increased at 12 and 16 weeks (Scott et al., 2007). Dahlgren et al. reported a 40% decrease in IGF-1 protein levels during the early stages of tendon healing compared to intact tendons, with levels peaking at 4 weeks and remaining elevated through 8 weeks post-injury (Dahlgren et al., 2005). In a rabbit patellar tendon model, Lyras et al. found that IGF-1 expression peaked at week three and gradually declined thereafter. Notably, IGF-1 expression was predominantly localized in inflammatory and endothelial cells during the first 2 weeks, shifting to tenocytes in the later stages of healing (Lyras et al., 2010). Another study using a rat flexor tendon transection-and-repair model demonstrated significantly elevated IGF-1 mRNA and protein levels at weeks four and eight compared to controls (Tang et al., 2019). Similarly, Berglund et al. observed a modest increase in IGF-1 mRNA levels by the end of the first week in a rabbit flexor tendon repair model, which surged nearly six-fold by week three and remained elevated at week 6 (Berglund et al., 2011).

In the single-time-point study, Tsubone et al. investigated IGF-1 expression in a canine flexor tendon repair model at 10 days post-operation using immunohistochemical staining, revealing a significant increase in IGF-1 levels (Tsubone et al., 2004). Kobayashi et al. reported elevated IGF-1 expression at day 5 in a rabbit supraspinatus tendon healing model (Kobayashi et al., 2006).

Although the timing of IGF-1 upregulation varies across studies, likely due to differences in species, experimental methods, and tendon locations, the consensus is that IGF-1 expression is significantly elevated during tendon healing. These findings deepen our understanding of the relationship between IGF-1 and tendon repair and provide a foundation for optimizing IGF-1 delivery strategies to enhance healing outcomes.

## 6 IGF-1 in tendon healing: experimental studies

Numerous *in vivo* studies using animal models have demonstrated the therapeutic benefits of local IGF-1 treatment in tendon healing (Table 1).

**TABLE 1:** The effect of IGF-1 in tendon healing: experimental studies.

Study	Animal models establish	Delivery strategy	Dosage	Time post operation	Outcome
Kurtz et al. (1999)	The Achilles tendons were transfected	Injection	Recombinant human IGF-1: 25 μg	15 days	Maximum functional deficit ↓ Synthesis of DNA↑ Collagen↑ Proteoglycans↑ Biomechanical failure loads →
Dahlgren et al. (2002)	A collagenase-induced model of flexor tendinitis	Injection	Recombinant human IGF-1: 0.2 μg rh IGF-1 injections every other day for 10 injections into the injured site	at 0, 2, 3, 4, 6, and 8 weeks	Lesion size ↓ Swelling ↓ Cell proliferation ↑ Collagen content ↑ Mechanical characteristics↑
Li et al. (2018)	The Achilles tendons were transfected and repair	used the clay nanoparticles to provide large surface areas to adsorb rhIGF-1 and assembled into micro particles that are physically trapped within the toughen hydrogel networks, providing extended and steady release of IGF-1	Recombinant human IGF-1 : 8 mg/mL	at 2 weeks	Biomechanical properties↑ Histological analysis ↑
Prabhath et al. (2022)	The supraspinatus tendon of rat was cut and repaired	Injection	Encapsulated pegylated IGF-1 mimic	At 8 weeks	Tensile properties↑ Fibrotic scar tissue↓
Dines et al. (2007)	The supraspinatus tendon of rat was cut and repaired	Gene delivery by retroviral vector	—	At 6 weeks	Toughness ↑ Maximal load to failure↑ Cellularity ↑ Well-organized collagen bundles↑
Tang et al. (2015)	The Achilles tendons were transfected and repair	Gene delivery by ultrasound-targeted microbubble destruction	—	At weeks 2 and 8	Inflammatory cells↑ Scar tissue↓ Maximum load↑ Stiffness ↑ Ultimate stress↑

Kurtz et al. investigated the effects of IGF-1 in a rat Achilles tendon transection model. Rats were divided into three groups: sham surgery, transection alone, and transection with recombinant human IGF-1 (rhIGF-1) injection (25 μg). The results revealed that IGF-1 enhanced DNA, collagen, and proteoglycan synthesis, accelerating functional recovery by day 15. However, no significant improvement in tendon biomechanical failure loads was observed compared to untreated controls (Kurtz et al., 1999).

Following the above study, Dahlgren et al. explored IGF-1’s effects in a collagenase-induced equine flexor tendon injury model. Equines were divided into a control group (0.9% NaCl injections) and an experimental group (0.2 μg rhIGF-1 injections every other day for 10 doses). Evaluations at 0, 2, 3-, 4-, 6-, and 8-week post-treatment demonstrated that the rhIGF-1-treated group exhibited increased cell proliferation, improved healing quality, enhanced collagen content, reduced tissue swelling, and superior mechanical properties compared to controls (Dahlgren et al., 2002).

Furthermore, in a rat model of Achilles tendon injury, Li et al. developed an innovative drug delivery system utilizing clay nanoparticles to enhance rhIGF-1 adsorption through their large surface area. These nanoparticles were subsequently assembled into microparticles and physically entrapped within a toughened hydrogel network. The hydrogel system was fabricated through a facile approach that combined clay nanoparticle incorporation, biodegradable hydrogel formation, and lyophilization treatment, achieving sustained and controlled release of rhIGF-1 at a concentration of 8 mg/mL. Notably, the IGF-1-treated group demonstrated significantly enhanced tendon biomechanical properties compared to the control group without growth factor treatment. Histological analysis further confirmed substantial improvement in tendon healing quality after 2 weeks of treatment (Li et al., 2018).

Despite its therapeutic potential, rhIGF-1 encounters several clinical limitations, primarily due to its structural homology with insulin, which may lead to hypoglycemia and growth hormone (GH) suppression (Jozefiak et al., 2021). To overcome these challenges, researchers have developed pegylated IGF-1, which preserves anabolic activity while mitigating adverse effects through reduced ligand-receptor interaction kinetics (Liang et al., 2022). Furthermore, rhIGF-1’s therapeutic application is constrained by its short *in vivo* half-life and susceptibility to proteolytic degradation. In comparison, chemically modified IGF-1 mimics demonstrate superior stability profiles. To evaluate these advantages, Prabhath et al. conducted a comprehensive study examining the effects of pegylated IGF-1 mimics in a rat supraspinatus tendon model. The researchers encapsulated both pegylated IGF-1 mimics and control formulations (unpegylated IGF-1 mimics and rhIGF-1) within polycaprolactone-based matrices, enabling localized and sustained delivery to the repaired tendons. The experimental results demonstrated that pegylated IGF-1 mimic treatment significantly reduced fibrotic scar formation, enhanced tendon structural regeneration, and improved biomechanical properties at 8 weeks post-treatment compared to control groups (Prabhath et al., 2022).

Gene delivery offers a promising alternative by introducing exogenous genetic material into cells to modulate protein expression. This approach ensures natural protein synthesis by host cells, avoiding reduced bioactivity and immune responses associated with exogenous biomolecule delivery (Jin et al., 2022; Ling et al., 2023). It also enables long-term protein release, reducing the need for repeated injections (Boye et al., 2022). Dines et al. demonstrated the efficacy of IGF-1 gene delivery in a rat rotator cuff tendon repair model. Rat tendon fibroblasts were transduced with an IGF-1 gene *via* a retroviral vector and seeded onto a bioabsorbable PGA scaffold. After 6 weeks, the gene-modified group exhibited improved toughness, maximal load to failure, and organized collagen bundles compared to controls (Dines et al., 2007). Tang et al. utilized ultrasound-targeted microbubble destruction (UTMD), a novel gene delivery strategy with high transfection efficiency and low immunogenicity, to deliver IGF-1 cDNA into rat Achilles tendons (Zhang et al., 2021). UTMD-mediated IGF-1 transfection enhanced inflammatory cell infiltration, reduced scar tissue formation, and improved maximum load, stiffness, and ultimate stress at weeks 2 and 8 (Tang et al., 2015).

Collectively, these studies highlight the significant therapeutic potential of IGF-1 in tendon healing across various animal models. However, challenges such as metabolic side effects and short half-life necessitate innovative delivery strategies, including Pegylation and gene therapy, to optimize its clinical application.

## 7 The mechanisms of IGF-1 for enhancing tendon regenerative healing

**FIGURE 3:**
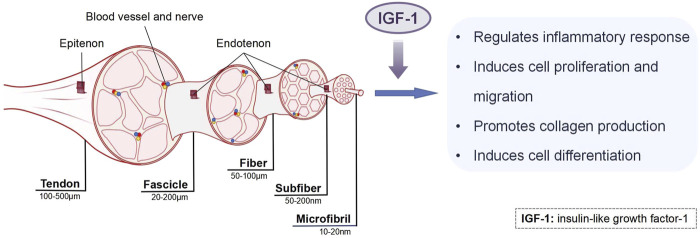
The mechanisms of IGF-1 in tendon healing.

Although the aforementioned studies demonstrate the therapeutic potential of IGF-1 in promoting tendon regenerative healing *in vivo*, the underlying mechanisms remain incompletely understood. To address this knowledge gap, *in vitro* cell and tissue culture studies have been conducted to elucidate the biological mechanisms by which IGF-1 enhances tendon repair. These investigations reveal that IGF-1 facilitates tendon regenerative healing through multiple pathways, including modulation of inflammatory responses, promotion of cell proliferation and migration, enhancement of collagen production, and induction of cell differentiation (Figure 3). These mechanisms will be discussed in detail in the following sections. However, it is important to note that the efficacy of IGF-1 is highly dependent on its dosage, which significantly influences its biological effects.

### 7.1 IGF-1 modulates inflammatory response

On the one hand, the inflammatory response, as the first stage of tendon healing, is necessary for injury tissue regenerative healing through killing pathogens and removing cell debris to prevent wound infection (Ribitsch et al., 2021). On the other hand, persistent inflammation inflicts scar tissue formation, resulting in poor remodeling and seriously hampers tendon regenerative healing (Wang et al., 2019b). During this phase, various inflamed cells invade the injured site and secreted several growth factors, mainly including IGF-1, VEGF, platelet-derived growth factor (PDGF), and TGF-β (Chartier et al., 2021). These growth factors diffuse into surrounding tissues and chemotactically attract neutrophils and monocytes into the damaged site. Subsequently, the monocytes differentiate into macrophages that regulate various healing processes (Huang et al., 2020). In fact, during the progression of the inflammatory process, macrophages have two different functional phenotypes, the M1 and M2 phenotypes (Wang et al., 2024). M1 macrophages are mainly activated by the innate immune system and are responsible for amplifying inflammatory reactions, while M2 macrophages are associated with the resolution of inflammation and tissue healing (Chen et al., 2021; Yang et al., 2022). Based on this, it has been proposed that enhancing the polarization of M1 to M2 macrophages could accelerate tendon regenerative healing. Potentially, the presence of IGF-1 in tendon regenerative healing has been reported to be associated with the activation of M2 macrophages (Prabhath et al., 2018). However, how IGF-1 affects M2 macrophages remains unknown. Further, in a study conducted by Kurtz et al., the Achilles tendons of 20 rats were transected, followed by an injection of the proinflammatory agent carrageenan. Subsequently, rhIGF-1 was applied to treat the injured site, and functional and biomechanical data were collected. It was found that the rats that received IGF-1 had a much less functional deficit induced by the carrageenan than rats that did not receive IGF-1, which suggests that IGF-1 may suppress the inflammatory responses during tendon healing (Kurtz et al., 1999).

Altogether, IGF-1 thus have the ability to both promote and dampen inflammatory response, suggesting a complicated regulatory influence on tendon healing. However, the mechanisms by which IGF-1 plays in the inflammatory response have been mostly elusive.

### 7.2 IGF-1 facilitates cell proliferation and migration

The limited number of resident tendon cells is one of the important reasons for the limited inherent regeneration ability of tendons (Chartier et al., 2021). Therefore, it is postulated that increasing the number of tenocytes within the injured site allows better tendon healing.

Several studies have confirmed that IGF-1 can enhance tenocyte proliferation and migration. For example, Costa et al. conducted a study to explore the effect of IGF-1 on three different cell populations of rabbit flexor tendons: synovial sheath, epitenon, and endotenon. Cell cultures were supplemented with IGF-1 (10, 50, and 100 ng/mL), and the results showed that cell proliferation was more significant in all cell types after IGF-1 stimulation compared to the control group (received no IGF-1). Moreover, the cells from the synovial sheath displayed a positive dose-response curve, whereas epitenon and endotenon were not dose-dependent and the highest values were reached at 10 and 100 ng/mL, respectively (Costa et al., 2006). These results suggest that the effect of IGF-1 on cell proliferation is cell-specific. Similar findings with IGF-1 (10, 50, and 100 ng/mL) treatment were noted using human tenocytes *in vitro*, leading to increased cell proliferation. However, this effect is not dose-dependent, and the highest values were reached with 50 ng/mL on day 3 after IGF-1 administration (Raghavan et al., 2012). A further study reported that tenocytes stimulated with IGF-1 (100 ng/mL) for 7 days exhibited a significantly increased cell proliferation compared to the tenocytes in cell groups cultured without IGF-1 (Durgam et al., 2012). Moreover, investigations on tenocytes of genetically modified mice further confirmed the effect of IGF-1 on cell proliferation. Given that many tenocytes expressing the gene scleraxis (SCX) play critical roles during tendon healing (Best et al., 2021), the researchers conditionally deleted the IGF-1R in SCX-expressing tenocytes by using a tamoxifen-inducible Cre-recombinase system. It was observed that mice that lack IGF-1R in their tenocytes displayed reduced cell proliferation compared to the control Scx-expressing IGF-1R-positive mice, suggesting that IGF-1 may have a role in enhancing cell proliferation. More importantly, it was also revealed that IGF-1 exerts its cell-proliferation-promoting effect through activating the PI3K the PI3K/protein kinase B and ERK pathways (Disser et al., 2019). Additionally, Caliari et al. also examined the effect of IGF-1 (10, 50, and 200 ng/mL) on equine tenocytes seeded on collagen-glycosaminoglycan scaffolds after 7 days. The results showed a dose-dependent effect of IGF-1 on tenocyte migration and proliferation compared to the non-supplemented group (Caliari and Harley, 2013). Separate extracts from epitenon and the internal compartment of avian flexor tendons can stimulate tenocyte DNA synthesis (mitotic activity); DNA synthesis was inhibited by an anti-IGF-1 antibody, suggesting that IGF-1 was the primary factor responsible for the extract’s mitogenic activity (Abrahamsson et al., 1991; Abrahamsson, 1997).

Overall, these results demonstrated the pivotal role of IGF-1 in enhancing cell proliferation and migration. However, limited studies have been conducted to understand the complete mechanisms of IGF-1 involved in cell proliferation and migration. Therefore, further research is warranted to examine the underlying mechanisms by which IGF-1 promotes cell proliferation during tendon healing.

### 7.3 IGF-1 accelerates collagen production

During tendon healing, there is a unique pattern of collagen production. In the proliferative phase, type III collagen increases significantly to provide a “quick fix” for the injured site (Dietrich-Zagonel et al., 2021). During the remodeling phase, the tendon tissue replaces type III collagen with type I collagen to bring structure and strength to the damaged site (Maeda et al., 2022). Thus, of particular relevance considering that type III and type I collagen are pivotal for the healing processes of damaged tendons, there are several documentations suggesting a critical role for IGF-1 in the regulation of collagen production.

Rat tenocytes cultured with IGF-1 (5, 10, and 20 ng/mL) for 3 days displayed different responses. The 10 ng/mL of IGF-1 remarkably increased collagen production, while the 10 and 20 ng/mL of IGF-1 treatment did not significantly increase collagen production compared to the untreated cells (Musson et al., 2017). In another study using rabbit Achilles tenocytes, the administration of IGF-1 led to the increased expression of type I collagen gene at 0.1 and 1 ng/mL, followed by a decrease at 10 ng/mL (Rieber et al., 2023). Moreover, experiments from tendon explants of equine also confirmed the positive effect of IGF-1 on ECM production in horse tenocytes, as the administration of 250 mg/mL IGF-1 resulted in an increased collagen production (Dahlgren et al., 2001). Furthermore, it has been reported that tenocytes with IGF-1 (10, 50, and 200 ng/mL) displayed the increased expression of tenocyte-associated structural protein genes for type I collagen (COL1A2), and type III collagen (COL3A1), but this effect is not dose-dependent and highest values were achieved at 50 ng/mL (Caliari and Harley, 2013).

Furthermore, several studies examined the effect of IGF-1 on human tendons. For example, after systemic administration of GH for 14 days, human data have reported an increase in circulating IGF-1 and expression of IGF-1 in tendon tissue, accompanied by increased expression of tendon collagen in individuals (Vestergaard et al., 2012). This study suggests that IGF-1 may play a role in promoting collagen production in humans, but neither provides any direct proof for a causative effect nor is it able to identify whether IGF-I or rather GH is the regulating factor for collagen synthesis in human tendons. Subsequently, a stimulating effect of IGF-1 on collagen tissue in human tendon tissue is supported by *in vivo* studies, which have demonstrated that local administration of IGF-1 (1 mg) directly induced an elevation in collagen synthesis in human tendon tissue (Hansen et al., 2013). Moreover, Nielsen et al. reported that patients respond to IGF-1 injection (10 mg/mL), leading to an increased collagen production of patellar tendon tissue (Nielsen et al., 2024). Consistently, another study also confirmed these previous experiments by showing a main effect of 250 ng/mL IGF-1 to increase collagen production in 3D and different FBS enriched media, where the constructs were divided into four treatment groups: (1)0.5% FBS, (2) 0.5% FBS+250 ng/mL IGF-1, (3) 10% FBS, (4) 10% FBS+250 ng/mL IGF-1. The results showed that in both IGF-1 treated-group, collagen was significantly increased at day 28 compared to non-IGF-1 supplementation groups (Herchenhan et al., 2015).

The above findings combined to demonstrate that IGF-1 is sufficient to increase collagen production in tenocytes. However, the underlying mechanisms of IGF-1-induced collagen production still remain elusive and therefore warrant further investigations.

### 7.4 IGF-1 induces cell differentiation

Current regenerative research has focused on the studies of growth factor supplementation that can induce stem cells into tenogenic lineage for an alternative cell source to replenish functional tendon cells at the injured site (Zhang et al., 2018). Mesenchymal stem cells (MSCs) and tendon stem/progenitor cells (TSPCs) have received increasing attention toward tenogenic differentiation and tendon regeneration (Wang et al., 2022). IGF-1 has been advocated to induce tenogenic differentiation of stem cells. For example, rat TDSCs treated with IGF-1 (1, 10, and 100 ng/mL) for 28 days exhibited the increased expression of tendon-specific marker (SCX) and significantly decreased expression of chondrogenic and osteogenic marker (collagen type II) (Holladay et al., 2016). Moreover, treatment with 10 ng/mL of IGF-1 for 10 days was demonstrated to be sufficient to induce tenogenic differentiation of bone marrow MSCs in 3D *in vitro* culture with significant upregulation of tenocytic markers (SCX, collagen type III, and decorin) (Durgam et al., 2012). These studies showed that IGF-1 can induce tenogenic differentiation of TSPCs and bone marrow MSCs. However, to date, no *in vivo* data are available regarding the efficacy of exogenously delivery of IGF-1 in combination with stem cells to treat tendon injury. Thus, future research is needed to determine the effect of the combination of IGF-1 and stem cells that leads to a regenerated tissue mimicking the intact tendon.

## 8 Future challenges and directions

Although numerous studies have demonstrated the potential of IGF-1 in advancing tendon treatment strategies, significant challenges remain in developing an effective IGF-1-based therapy for tendon regenerative healing.

One major limitation is the short half-life of growth factors under physiological conditions. When directly injected, IGF-1 rapidly diffuses away from the injury site, making it difficult to maintain therapeutically effective concentrations (Caballero Aguilar et al., 2019). Consequently, high dosages or repeated injections are often required, which not only increase treatment costs but also raise the risk of adverse effects (Muller et al., 2015). These limitations underscore the need for advanced delivery systems that can localize and sustain IGF-1 at the injury site (Yang et al., 2021). While gene therapy and tissue-engineered scaffolds offer promising alternatives (as discussed earlier), further optimization of these platforms is essential for successful clinical translation.

Secondly, despite its promising results *in vitro* and in animal models, clinical trials have not consistently shown a significant role for IGF-1 in promoting tendon repair (Olesen et al., 2021). This discrepancy could be attributed to multiple factors. In clinical practice, patients are a heterogeneous group with diverse genetic backgrounds, lifestyles, and underlying health conditions. These individual differences may influence the body’s response to IGF-1. For example, genetic polymorphisms in the genes encoding IGF-1 receptors or components of its signaling pathway may lead to variations in the effectiveness of IGF-1 therapy (Cubbon et al., 2016). Some patients may have mutations or polymorphisms that result in reduced receptor affinity for IGF-1 or impaired downstream signaling, thus limiting the therapeutic effect (Weroha and Haluska, 2008). Additionally, the complex *in vivo* environment in humans is difficult to replicate in laboratory settings. Inflammation, immune responses, and the presence of other biological factors in the human body can interact with IGF-1 in ways that are not fully understood, potentially dampening its efficacy.

Another critical area requiring further investigation is the optimization of IGF-1 dosage, which is essential for its successful application in tendon injury treatment. As previously noted, studies have employed varying dosages, such as 25 μg, 0.2 μg, and 8 mg/mL, in animal models, yielding inconsistent effects on tendon regeneration. For instance, a high dosage of IGF-1 (100 ng/mL) was found to be detrimental in a mouse study, inducing ectopic ossification and impairing Achilles tendon healing. Conversely, lower dosages have demonstrated therapeutic potential in promoting tendon repair in animal models (Mao et al., 2024). However, translating these findings to human patients presents significant challenges due to the complexity of human physiology, including factors such as body size, metabolic rate, and individual variability. Additionally, the optimal dosage may vary depending on injury-specific factors, such as location, severity, and chronicity. Therefore, future research should focus on establishing standardized dosing protocols through well-designed preclinical and clinical studies, considering both biological and injury-related variables to maximize the therapeutic efficacy of IGF-1 in tendon repair.

Additionally, given the complex and multifaceted nature of tendon healing, it is likely that delivering combinations of growth factors will result in the most beneficial outcomes for tendon regenerative healing. For example, Xiang et al. used UTMD to transfect IGF-1 cDNA and TGF-β short hairpin RNA (shRNA) into a rat model of Achilles tendon repair. They found that the combination of these growth factors displayed a more significant maximum load and tensile stress and inhibited scar formation compared to the IGF-1 alone group, suggesting that combination of IGF-1 and TGF-β is more effective than IGF-1 alone in the treatment of tendon injury (Xiang et al., 2018). Thus, future research is needed to determine the optimum combination of IGF-1 and other growth factors, making full use of the synergistic effects of various growth factors to induce their therapeutic potential and, thus, better regulate tendon healing.

Moreover, the efficacy of IGF-1 therapy can be influenced by factors such as age, health conditions, and injury type. In aged tendons, tenocyte activity is impaired, and functionally compromised pro-inflammatory tenocytes accumulate (Korcari et al., 2023). These age-related alterations may disrupt IGF-1 signal transduction, hinder downstream signaling, and reduce tenocyte responsiveness, ultimately impairing tendon repair and regeneration. Similarly, health conditions such as obesity can alter the therapeutic outcome, as the pro-inflammatory microenvironment characterized by elevated cytokine levels in obese patients may interfere with IGF-1 signaling and impede tendon healing (Hjelholt et al., 2020). Furthermore, the type of tendon injury plays a critical role in determining the treatment response. While IGF-1 promotes cell proliferation and collagen synthesis in acute partial tears, its effectiveness in chronic tendinopathy is often limited due to persistent inflammation, tissue degeneration, fibrosis, and abnormal ECM composition, which collectively restrict IGF-1 diffusion and reduce cellular responsiveness. The response to IGF-1 treatment may vary depending on the type of tendon injury. In acute partial tears, IGF-1 has been shown to enhance cell proliferation and collagen synthesis (Miescher et al., 2023). However, in chronic tendinopathy, its efficacy may be compromised due to persistent inflammation, tissue degeneration, fibrosis, and abnormal ECM composition, which can impede IGF-1 diffusion and diminish the responsiveness of tendon cells (Tang et al., 2024). These adaptations emphasize that IGF-1 therapy cannot follow a universal protocol, but rather requires multidimensional optimization based on individual patient profiles and injury context. Future clinical trials should incorporate stratified randomization based on these key modifiers of treatment response.

## 9 Conclusion

Tendon injury is a major musculoskeletal disorder that causes pain, limited activity, significant disability, and lost productivity. Following a tendon injury, the tendon tissue fails to regenerate, often forming scar tissue. Currently, various therapeutic strategies for tendon injury are available, including conservative and surgical treatments. However, these treatments are not effective. Therefore, there is an urgent need to develop novel treatments for tendon injury. Over the last 2 decades, IGF-1 has been discovered providing a positive scientific basis for its use in treating tendon disorders. This review highlights the multifaceted role of IGF-1 in promoting tendon regenerative healing, including its ability to modulate inflammatory responses, stimulate cell proliferation and migration, and enhance ECM production. However, to fully realize its therapeutic potential, critical factors such as optimal dosage, timing of application, and delivery strategies must be further investigated and refined.
